# Altered Expression of MeCP2 and PTEN Genes in the Molecular Basis of Specific Learning Disorder

**DOI:** 10.1007/s12031-025-02370-3

**Published:** 2025-06-06

**Authors:** Fatma Atasever, Nil Özbilüm Şahin, Cansu Mercan Işık

**Affiliations:** 1 Department of Molecular Biology and Genetic, Faculty of Science, Sivas Cumhuriyet University, 58140 Sivas, Turkey; 2 Department of Child and Adolescent Psychiatry, Faculty of Medicine, Sivas Cumhuriyet University, Sivas, Turkey

**Keywords:** Specific Learning Disorders, MeCP2, PTEN, Gene Expression, QPCR

## Abstract

Specific learning disorders (SLD) are neurodevelopmental disorders that affect cognitive abilities such as reading, writing, and mathematics. The molecular mechanisms underlying SLD remain unclear, though genetic and epigenetic factors are thought to play a significant role. MeCP2 is an epigenetic regulator that binds to methylated DNA, playing a crucial role in the regulation of gene expression and SP in neuronal cells. PTEN, a tumor suppressor gene, regulates cell growth, survival, and apoptosis, and is critical for maintaining synaptic integrity. In this study, we aimed to examine the expression of MeCP2 and PTEN in individuals with SLD. RNA was isolated from blood samples, and gene expression was assessed using quantitative PCR (qPCR). A total of 38 participants with SLD and 35 healthy controls were included in the study. Our results revealed a 15.44-fold upregulation of MeCP2 and a 13.66-fold downregulation of PTEN in the SLD group compared to controls, suggesting a disrupted balance of gene expression. There was no significant difference in gene expression between severe and non-severe SLD groups. These findings suggest that the dysregulation of MeCP2 and PTEN may be involved in the pathophysiology of SLD, influencing SP and neuronal function. In conclusion, the altered expression of these genes in individuals with SLD highlights potential biomarkers for early diagnosis and therapeutic targets, opening avenues for future research and intervention strategies.

## Introduction

Specific Learning Disorder (SLD) is a neurodevelopmental disorder (ND) characterized by significant difficulties in learning and using academic skills, which are not consistent with the individual’s age and educational opportunities. According to the Diagnostic and Statistical Manual of Mental Disorders, Fifth Edition (DSM-5), SLD encompasses various specific impairments, including dyslexia (reading difficulties), dysgraphia (writing difficulties), and dyscalculia (mathematical difficulties) (Sanfilippo et al. 2020). These disorders manifest as a failure to acquire and use academic skills, leading to academic underachievement and functional impairments in daily life (Fortes et al. 2016). The prevalence of SLD is estimated to affect approximately 5–15% of the school-age population, highlighting the importance of early identification and intervention (Fortes et al. 2016). The impact of SLD on academic performance is profound and multifaceted, as children with SLD often face significant challenges in core academic areas, including reading, writing, and mathematics, which hinders their overall educational progress (Sahoo et al. 2015). Beyond academics, these difficulties can impair social interactions and self-esteem, contributing to a cycle of frustration and disengagement from the learning process. Additionally, children with SLD are at a higher risk of developing comorbid conditions, such as anxiety and depression, which exacerbate their academic struggles (Margari et al. 2013).

In this study, we aimed to explore the differential expression of Methyl-CpG-binding protein 2 (MeCP2) and The phosphatase and tensin homolog (PTEN) genes in patients with SLD, compared to a control group. We hypothesized that these two genes, which have been implicated in various ND, may also play a role in the pathophysiology of SLD. MeCP2 plays a critical role in synaptic scaling, a homeostatic process that adjusts synaptic strength in response to neuronal activity. Research indicates that the absence of MeCP2 disrupts synaptic scaling, leading to significant impairments in synaptic function and plasticity (Zhong et al. 2012; Blackman et al. 2012). Furthermore, MeCP2 functions as a transcriptional regulator, which is essential for maintaining neuronal function and synaptic integrity (Nguyen et al. 2012; Zhong et al. 2012). In our previous studies, epigenetic changes were observed in some genes associated with SLD, but the role of high-level regulators such as MeCP2 and PTEN has not yet been elucidated (Bayyurt et al. 2024; Isik et al. 2025).

PTEN is a crucial regulator of neuronal growth, development, and neuroprotection. It acts primarily as a lipid phosphatase that inhibits the phosphoinositide 3-kinase PI3 K/AKT/mTOR signaling pathway, essential for neuronal survival and growth (Liu et al. 2021; Xu et al. 2024). PTEN plays a critical role in maintaining proper neuronal morphology, synaptic plastic (SP), and overall brain function. PTEN has been shown to inhibit excessive neuronal growth, which is critical for proper axonal and dendritic development (Baohan et al. 2016; Kath et al. 2018). Following activation of the mTOR signaling pathway, there is an increase in the synthesis of presynaptic proteins that are crucial for synaptic plasticity processes, the formation and maturation of new dendritic spines, memory processes or long-term potentiation (LTP) (Garro-Martínez et al. 2021). The Akt/mTOR pathway is crucial for the process of learning and memory formation by enhancing LTP of synapses (Opazo et al. 2003). PTEN mutations are linked to clinical syndromes, including autism spectrum disorder (ASD) (Spinelli et al. 2015; Lyu et al. 2019). PTEN knockout mice develop behavioral abnormalities suggestive of human autism, with reduced learning and social interaction deficits (Clipperton-Allen and Page 2014; Busch et al. 2019). Neurobehavioral assessments conducted in individuals with PTEN-related ASD indicate that severe cognitive dysfunction, slow reaction time, attention deficits, and reduced memory processing capacity are primarily associated with impairments in frontal lobe systems (Clipperton-Allen and Page 2014). Additionally, PTEN has a significant neuroprotective role by regulating cellular responses to stress, including neurotoxic insults. Its downregulation has been associated with increased resistance to neurotoxin-induced cell death, highlighting its role in cellular survival mechanisms (Kwak et al. 2010). MeCP2 and PTEN are critical regulators of neurodevelopmental processes including synaptic plasticity, neuronal maturation, and migration. Given their involvement in these processes, it is hypothesized that dysregulation of MeCP2 and/or PTEN may impair learning by altering the structure and function of neural circuits responsible for academic skill acquisition, particularly within regions like the hippocampus and prefrontal cortex. Such dysregulation could lead to deficits in long-term potentiation, dendritic arborization, and network synchrony, ultimately contributing to the core features of SLD. Therefore, the aim of this study was to investigate the potential role of MeCP2 and PTEN gene expressions in the pathophysiology of Specific Learning Disorder (SLD), with a focus on understanding their possible contributions to the molecular mechanisms underlying this ND.

## Methods

### Study Design

This study was conducted as an interdisciplinary study with the Departments of Child Psychiatry, Medical Biology and Molecular Biology and Genetics of Sivas Cumhuriyet University Faculty of Medicine. Ethical approval was obtained from the Sivas Cumhuriyet University Non-Interventional Clinical Research Ethics Committee to conduct the study and collect blood from individuals (Decision No: 2024–07/46, decision date: 18.07.2024). The study consisted of four main stages: collection of blood samples from individuals, RNA isolation from the blood samples qPCR analysis after cDNA synthesis, and statistical analysis.

### Study Participants

This study included a total of 73 participants, consisting of 38 children and adolescents diagnosed with SLD and 35 healthy controls. Participants were recruited from the Child and Adolescent Psychiatry Department of the Faculty of Medicine at Sivas Cumhuriyet University. Children and adolescents aged between 6 and 16 years who were diagnosed with SLD and applied to the Child and Adolescent Psychiatry outpatient clinic were included in the study. The control group consisted of 35 children and adolescents with no psychiatric disorders, matched with the SLD group in terms of demographic characteristics. The study included 73 participants (35 control and 38 SLD), with an *α* of 0.05, *β* of 0.10, and a power of (1-*β*) = 0.90, resulting in a statistical power of *p* = 0.90718. The following criteria were used to create the groups: age between 6 and 16 years, no other mental illnesses (other than SLD) in the SLD group, no mental illness in the control group, no history of head trauma or neurological disease, no participation in a similar study within the past 6 months, and no use of medications that could potentially affect cognitive processes. Individuals who did not meet these criteria were excluded from the study group. Blood samples of individuals were collected in RNA Stabilizer Tube (NucleoGene, NG20200803). Blood samples collected in RNA tubes were stored at − 20 °C until RNA isolation.

### Neuropsychological Measures and Clinical Assessment

To gather information on the participants’ sociodemographic characteristics, a researcher-designed form including variables such as age and sex was utilized. Psychiatric evaluations of all children and their parents were conducted using the Turkish version of the semi-structured Kiddie Schedule for Affective Disorders and Schizophrenia for School-Age Children–Present and Lifetime Version (K-SADS-PL) (Kaufman et al. 1997; Ünal et al. 2019). In order to confirm a diagnosis of combined-type pure Specific Learning Disorder (SLD), participants underwent a series of standardized assessments targeting reading, writing, and mathematical abilities. The Wechsler Intelligence Scale for Children – Fourth Edition (WISC-IV) was employed to evaluate general intellectual functioning (Grizzle 2011). Additionally, to rule out Attention Deficit Hyperactivity Disorder (ADHD) symptoms co-occurring with SLD, a revised short form of the Conners’ Parent Rating Scale-Revised: Short Form (CPRS-RS) was administered (Kaner et al. 2013).

### RNA Isolation from Blood Samples

Hybrid-R RNA isolation kit was used for total RNA isolation from blood samples (GeneAll, Cat. No.305–101). All isolation steps were performed according to the manufacturer’s recommendations with minor modifications. All details of the isolation steps were analyzed in detail in our previous studies (Bayyurt et al. 2024; Isik et al. 2025). The quality and quantity of isolated RNAs were measured using nanodrop as they are important in the next step, cDNA synthesis (Maestro, NANO).

### Complementary DNA Synthesis (cDNA) and Quantitative Real-Time PCR (qPCR)

The NucleoGene cDNA Synthesis Kit (5X) (NucleoGene, Cat. No: NGMM019) was used for cDNA synthesis, following the manufacturer’s recommendations. The LightCycler® 96 Real-Time PCR Instrument (Roche) was used to perform qPCR. The qPCR reaction was performed using the Nucleogene qPCR master mix according to the protocol recommended by the manufacturer. Nucleogene primer sequences were used for the MeCP2 and PTEN genes (NucleoGene Gene Expression Assay, NGOT005). In each qPCR reaction, 10 μl of qPCR mix, 2 μl of primer analysis, 2 μl of cDNA, and 6 μl of nuclease-free water were used in a total reaction volume of 20 μl. Expression values were normalized using GAPDH as the internal control gene. A melting curve analysis was performed for each primer in the qPCR step to confirm the specificity of the PCR amplification.

### Statistical Analysis

To assess differences in demographic parameters between the SLD and control groups, a chi-square (*χ*^2^) test was performed using SPSS (ver: 23.00) (Table 1). The *χ*^2^ test is commonly used to compare categorical variables and evaluate significant associations between two or more groups. In this study, it was applied to assess the distribution of demographic characteristics such as gender, age group, and other relevant factors between the SLD and control groups. A *p* value of less than 0.05 was considered statistically significant.

**Table 1:** Demographic parameters of child and adolescent participants

Variable	SLD group, ***n* (%)**	Control group, ***n* (%)**	*p* value
Sex			
Male	23(60.53)	18(51.43)	
Female	15(39.47)	17(48.57)	0.434
Age			
Mean (years ± SD)	10.26 ± 2.6	10.34 ± 2.6	0.892
Severity			
Severe	21(55.26)		
Non-severe	17(44.74)		
Living			
Province	29 (76.32)	29 (82.86)	
District	6 (15.79)	6 (17.14)	
Village	3 (7.89)	0(0.00)	0.237
Family structure			
Core	29(76.32)	31(88.58)	
Large	4(10.53)	2 (5.71)	
Parents divorced	3 (7.89)	0	
At least one of the parents is deceased	2 (5.26)	2 (5.71)	0.306
Disease during pregnancy			
No	36 (94.74)	34 (97.14)	
Yes	2 (5.26)	1 (2.86)	0.605
Drug use during pregnancy			
No	36 (94.74)	35 (100.00)	
Yes	2 (5.26)	0 (0.00)	0.494
Smoking during pregnancy			
No	33 (86.84)	26 (74.29)	
Yes	5 (13.16)	9 (25.71)	0.237
Type of birth			
Vaginal birth	30 (78.95)	28 (80.00)	
C/S	8 (21.05)	7 (20.00)	0.999
Time of birth			
Early	8 (21.05)	2 (5.71)	
Mid	27 (71.05)	32 (91.43)	0.084
Late	3 (7.90)	1 (2.86)	0.999
Birth weight			
Under 2500 g	5 (13.16)	2 (5.71)	
Between 2500 and 4000 g	30 (78.95)	31 (88.58)	0.429
Over 4000 g	3 (7.89)	2 (5.71)	0.999
Birth complications			
No	32(84.21)	32 (91.43)	
Yes	6 (15.79)	3 (8.57)	0.482

**Fig. 1:**
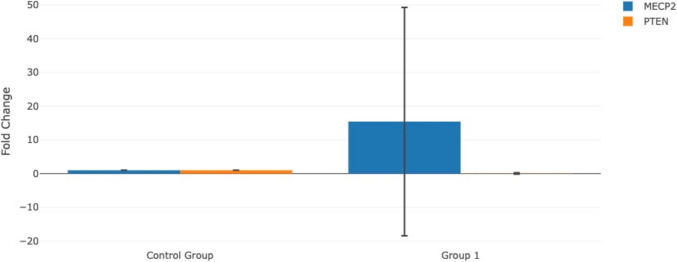
Fold change (FC) expression levels of MECP2 and PTEN in children with group 1compared to the control group. FC values were calculated using the 2^−ΔΔCt^ method, with GAPDH as the internal control gene for normalization. Group 1: SLD group

**Fig. 2:**
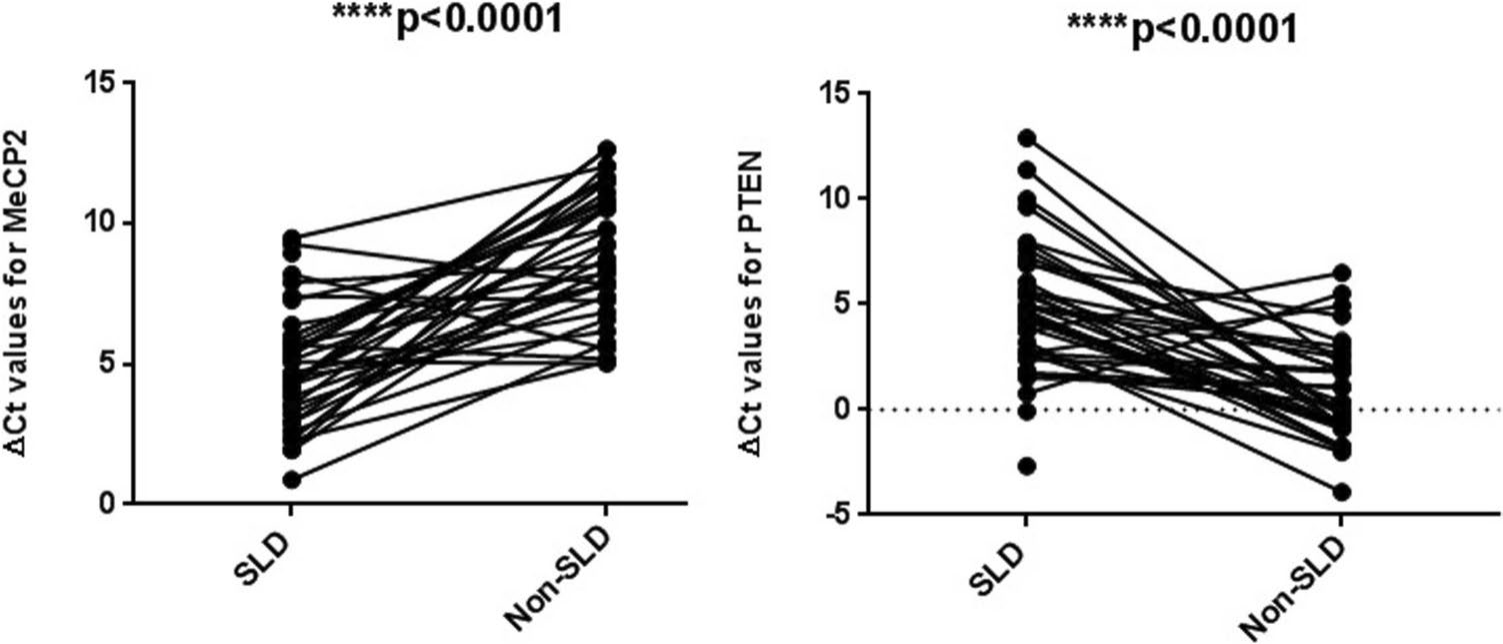
Comparison of individual ΔCt values of SLD and non-SLD groups. Each line represents an individual; ΔCt values of individuals under SLD and non-SLD conditions are shown. ΔCt values were calculated by subtracting the Ct value of the internal control gene (GAPDH) from the Ct value of each target gene (ΔCt = Ct_target − Ct_GAPDH). Paired plots represent matched samples, and statistical significance was assessed using a paired t-test. *p* < 0.05 was considered significant

**Fig. 3:**
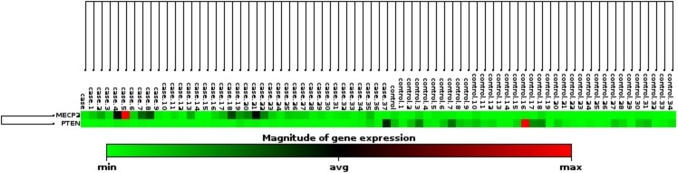
Heat map analysis of expression profiles of MeCP2 and PTEN genes in SLD and control groups. Heat map represents individual-based gene expression levels. Color intensity reflects normalized expression values. Low expression levels are shown in cool colors (e.g., blue), high expression levels are shown in warm colors (e.g., red). Data were grouped by hierarchical clustering among genes and/or samples. Expression patterns in SLD group were compared with control group

The Ct values obtained for MeCP2 and PTEN from qPCR were analyzed to determine whether there was a significant difference between the SLD and control groups. FC figures were created with this database (Fig. 1 and Table 2). GraphPad Prism (ver: 6.01) was used for Fig. 2 and statistical analysis (t-test). The expression level of MeCP2 and PTEN was normalized using the housekeeping gene GAPDH. The normalized expression levels were calculated using the fold change (FC) method and the relative expression levels of each gene were determined using the 2^−ΔΔCt^ (delta-delta Ct) method using the software “GeneGlobe Data Analysis Center” (https://geneglobe.qiagen.com/us/analyze). Clustergram figures were also created with this database (Fig. 3). Multiple regression analyses were performed to determine the effects of demographic and clinical data on gene expression. MeCP2 and PTEN gene expression levels were used as dependent variables, and demographic and clinical parameters such as gender, smoking during pregnancy, and delivery type were used as independent variables. All parameters are presented in Table 3. Linear regression model was applied for multiple regression analysis and separate analyses were performed for both gene expressions. The suitability of the models was evaluated with *R*^2^, and statistical significance was accepted at *p* < 0.05. Independent variables with variance inflation factor (VIF) values below 10 were included in the analysis as they were considered not to have high multicollinearity in the model. The SPSS 23.0 software was used in all analyses.

**Table 2:** Comparison of gene expressions between different groups

Comparison	FC/FR/p	Name of gene	
		MeCP2	PTEN
SLD vs. control	FC	15.44	− 13.66
	FR	15.44/upregulated	0.074/downregulated
	*p* value	**0.0002**	**0.0091**
Male vs. female	FC	− 1.08	1.32
	FR	0.92/downregulated	1.32/upregulated
	*p* value	0.089	0.082
Severe vs. non-severe	FC	− 1.43	− 1.24
	FR	0.70/downregulated	0.801/downregulated
	*p* value	0.48	0.871

*FC*, fold change; *FR*, fold regulation; *p* < 0.05 is statistically significant; *SLD*, specific learning disorder. Bold values ​​are statistically significant (*p*<0.05)

**Table 3:** Independent and dependent variables, and demographic and clinical parameters

Variables		MeCP2				PTEN		
	*B*	*p*	***β***	VIF	*B*	*p*	***β***	VIF
Sex	− 0.074	0.763	− **0.039**	1.147	− 0.214	0.780	− **0.023**	1.145
Drug use during pregnancy	− 1.435	0.098	− **0.250**	1.510	− 7.589	0.006	− **0.265**	1.496
Smoking during pregnancy	0.531	0.102	**0.223**	1.231	0.135	0.892	**0.011**	1.219
Radiation exposure during pregnancy	− 0.705	0.523	− **0.087**	1.264	− 4.541	0.186	− **0.113**	1.245
Type of birth	0.083	0.788	**0.036**	1.190	1.691	0.082	**0.146**	1.190
Birth complication	0.235	0.627	**0.082**	1.941	− 1.929	0.203	− **0.136**	1.941
Receiving incubator care	− 0.039	0.928	− **0.015**	1.910	0.539	0.684	**0.043**	1.910
Additional medical illness	0.601	0.246	**0.189**	1.774	1.721	0.278	**0.108**	1.715
History of speech disorders	0.058	0.861	**0.024**	1.318	0.725	0.465	**0.061**	1.209
Mental illness in father	0.032	0.924	− **0.160**	1.301	1.069	0.315	**0.065**	1.301
Mental illness in mother	− 0.404	0.284	**0.013**	1.490	0.819	0.474	**0.088**	1.421
Severity	− 0.005	0.961	− **0.007**	1.475	2.811	< 0.001	**0.790**	1.472

*VIF*, variance inflation factor, statistical significance; *p* < 0.05, B: unstandardized regression coefficient, β beta, standardized regression coefficient. Bold values ​​are statistically significant (*p*<0.05)

## Results

In this study, MeCP2 and PTEN gene expression study was conducted to compare the SLD and control groups, which consisted of 38 individuals with SLD and 35 healthy controls, making a total of 78 participants. Demographic parameters were tested with chi-square test in SPSS, and no significant differences were found between different groups. These findings are summarized in Table 1. We also showed all other data regarding demographic parameters in our previous study (Bayyurt et al. 2024). We also analyzed gene expression values based on sex and severity. In the SLD group, there were 23 males and 15 females, while the control group consisted of 18 males and 17 females (Table 1). No statistical differences were found between males and females in terms of demographic parameters. Furthermore, the SLD group was divided into severe and non-severe subgroups. Of the 38 individuals with SLD, 21 had severe SLD, while 17 had non-severe SLD. Other parameters, such as living area, family structure, and disease during pregnancy, are shown in Table 1.

For gene expression analysis, Table 2 presents the results for the MeCp2 and PTEN genes, where differences in gene expression between the SLD and control groups were examined using the Gene Globe Data Analysis Center. The analysis revealed that PTEN expression was downregulated by 13.66-fold (FC =  − 13.66, FR = 0.074, *p* = 0.0091) in the SLD group compared to the controls. In contrast, MeCP2 expression was upregulated by 15.44-fold (FC = 15.44, FR = 15.44, *p* = 0.00023) in the SLD group relative to the control group.

Additionally, we analyzed gene expression differences based on the severity of SLD. However, when we divided the SLD group into severe and non-severe subgroups, no significant differences were found between the two groups. Both MeCP2 and PTEN gene expressions were downregulated in both subgroups (MeCP2 FC =  − 1.08, FR = 0.92, *p* = 0.089; PTEN FC =  − 1.32, FR = 1.32, *p* = 0.082). A sex-based analysis was also conducted. In this analysis, MeCP2 expression was downregulated (FC =  − 1.43, FR = 0.70, *p* = 0.48), while PTEN was upregulated in both the SLD and control groups (FC =  − 1.24, FR = 0.801, *p* = 0.871). However, neither of these differences reached statistical significance. Figure 1 displays the FC graphs calculated using the Gene Globe Data Analysis Center.

Statistical differences between the Ct values of the SLD and control groups were determined using a t-test. The results were visualized using GraphPad software, as shown in Fig. 2. The analysis revealed that while the Ct values for the PTEN gene were statistically significant between the SLD and control groups (*p* < 0.0001), the Ct values for the MeCP2 gene were not statistically significant. It was observed that the expression level of the MeCP2 gene was particularly increased in the SLD group, whereas the expression level of the PTEN gene was higher in the control group. These differences suggest that the regulation of both genes is specifically linked to the disease state. In the heat map (Fig. 3), MeCP2 gene expression was highlighted in red tones, indicating high expression in some samples, while PTEN gene expression was represented in blue tones, showing lower expression. This color distribution clearly demonstrated the gene expression differences between the groups.

The study investigated the relationships between MeCP2 and PTEN gene expression and various demographic and environmental variables in SLD patients. According to regression analysis, significant relationships were found between PTEN gene expression and some variables. In particular, drug use during pregnancy (*B* = − 7.589, *p* = 0.006) and severity of disease (*B* = 2.811, *p* < 0.001) showed significant effects on PTEN expression levels. No significant effect of other variables was observed on both MeCP2 and PTEN expressions (*p* > 0.05). No statistically significant effect of any independent variable was found on MeCP2 gene expression (Table 3).

## Discussion

Recent research into genetic and epigenetic markers in SLD has revealed important insights into the mechanisms that contribute to cognitive impairments (Kramer 2013; Amador-Arjona et al. 2015). SLDs are often influenced by both genetic predispositions and environmental factors, an interplay referred to as “epigenetic” (Weiss et al. 2023). Differentiating SLD from other conditions, such as intellectual disabilities or ASD, is crucial because while these conditions may co-occur, they require distinct interventions support strategies (Petretto and Masala 2017; Sanfilippo et al. 2020) Additionally, the identification of SLD is often complicated by comorbidities such as Attention-Deficit/Hyperactivity Disorder (ADHD), anxiety, and mood disorders, which can exacerbate the learning difficulties (Narayanan et al. 2018).

The MeCP2 gene is well-known for its role in chromatin remodeling and gene repression, particularly in neuronal cells, where it binds to methylated DNA and regulates the transcription of genes essential for normal neuronal function (Mellén et al. 2012; Zhao et al. 2013; Nettles et al. 2023). Mutations in the MECP2 gene are associated with Rett syndrome (RTT), an ND, underscoring the importance of MeCP2 in maintaining normal cognitive functions (Wu and Li 2022). Conversely, PTEN is a tumor suppressor gene that plays a pivotal role in the regulation of cell growth and survival, and its dysregulation has been implicated in various ND, including ASD (Lyu et al. 2016). Specifically, Blackman et al. demonstrated that neurons lacking MeCP2 are unable to compensate for prolonged developmental loss, resulting in a failure to scale synaptic strength appropriately (Blackman et al. 2012). This loss of synaptic scaling is posited to be a major defect contributing to the pathophysiology of RTT. Furthermore, the phosphorylation of MeCP2 at specific serine residues is essential for its role in modulating synaptic scaling through metabotropic glutamate receptors (Zhong et al. 2012). In a study in RTT, post-translational modifications of MeCP2 were shown to regulate the expression of genes involved in synaptic formation, suggesting that a defective modification may contribute to the pathogenesis of RTT (Cheng et al. 2014). The importance of MeCP2 in synaptic development has been supported by its association with altered synaptic function in the amygdala (Gambino et al. 2010). The role of MeCP2 is also to influence the expression of several synaptic proteins that are essential for maintaining synaptic integrity. Loss of MeCP2 has been shown to reduce levels of essential synaptic proteins that are critical for excitatory synapse maturation. This reduction likely contributes to the observed retraction of dendritic arbors and synaptic dysfunction in RTT (Nguyen et al. 2012). Moreover, the dysregulation of microglial function associated with MeCP2 mutations has been implicated in synaptic damage, as microglia release elevated levels of glutamate, further exacerbating synaptic impairment (Maezawa and Jin 2010). The 15-fold increase in MeCP2 gene expression in SLD individuals indicates a unique pathophysiological mechanism that is different from the ND generally associated with MeCP2 deficiency in the current literature. MeCP2 is an epigenetic regulator that regulates gene expression and shapes SP by binding to methylated DNA regions (Mellén et al. 2012). Due to its dose-sensitive structure, both its deficiency and excess can lead to neuronal homeostasis. While MeCP2 loss in conditions such as Rett Syndrome causes a decrease in synaptic connections, it is thought that MeCP2 overexpression may excessively limit neuronal activity by increasing gene suppression. This may contribute to cognitive dysfunctions seen in SLD, especially by suppressing the expression of genes associated with learning and memory. Furthermore, considering the regulatory role of MeCP2 on synaptic scaling and protein expression, such an increase may prevent optimal learning processes by reducing the flexibility of SP. To better understand how MeCP2 upregulation contributes to the neurobiological mechanisms in SLD, it is of great importance to detail this increase by cell type, brain region, and target genes.

The role of PTEN in neuroprotection is multifaceted. PTEN is a tumor suppressor gene that plays a central role in the regulation of cellular growth, differentiation, and survival processes and is especially critical for balancing apoptosis in neurons. PTEN is involved in the cellular response to various stressors, including neurotoxic insults. Its activity is crucial for regulating apoptosis in neurons, as downregulation of PTEN has been associated with increased resistance to neurotoxin-induced cell death through the activation of the Akt survival signaling pathway (Kwak et al. 2010). Decrease of PTEN may result in uncontrolled activation of the PI3 K/AKT/mTOR signaling pathway, leading to effects such as excessive growth, synaptic disorganization, and neuronal hyperexcitability in neuronal cells (Kwak et al. 2010). The dysregulation of PTEN signaling can lead to altered synaptic development and plasticity, which are critical for normal cognitive and behavioral functions. For instance, the loss of PTEN in excitatory neurons has been linked to increased excitation and altered interneuron function, which may contribute to the hyperexcitability observed in ASD (Vogt et al. 2015; Gallent and Steward 2018).Furthermore, PTEN’s involvement in SP is essential for learning and memory processes. Research has shown that PTEN regulates synaptic density and morphology, influencing the formation and stabilization of synapses during development (Nolan et al. 2019; Benetatos et al. 2020). The loss of PTEN has been shown to cause a marked increase in dendritic arborization and a significant enlargement of the hippocampus. Additionally, PTEN deficiency disrupts synaptic plasticity by impairing both long-term potentiation (LTP) and long-term depression (LTD) mechanisms. In our study, the fact that PTEN gene expression was significantly decreased by approximately 14-fold in individuals with SLD indicates that neurodevelopmental processes may be impaired at the molecular level in these individuals (Table 2, Fig. 1). These changes may lead to disruption of SP, which is necessary for the healthy execution of higher cognitive functions such as learning and memory. Indeed, the neuroprotective function of PTEN has also been associated with its capacity to increase the resistance of neurons to various stressors and neurotoxic stimuli. In this context, decreased PTEN levels in individuals with SLD may be considered as a molecular basis for increased neuronal fragility and therefore cognitive dysfunction. In addition, PTEN depletion may cause abnormal neuronal growth or differentiation and may contribute to the explanation of SLD observed through synaptic structures. Genetic studies have identified mutations in the PTEN gene that are associated with ASD, highlighting its importance in neurodevelopmental processes (Spinelli et al. 2015; Vogt et al. 2015). PTEN genetic mutations are highly dominant in developmental delays and intellectual disabilities (Varga et al. 2009). Cognitive abnormalities have been observed in PTEN-ASD patients, with prominent white matter and reduced processing speed and working memory deficits (Frazier et al. 2015). The modulation of synaptic structures by PTEN is crucial for maintaining the balance between excitation and inhibition in neural circuits, which is often disrupted in ASD (Vogt et al. 2015; Gallent and Steward 2018). Deletion of the PTEN gene in hippocampal neurons results in the overactivation of the PI3 K-AKT/mTOR signaling cascade, a key regulator of neuronal development, synaptic plasticity, and memory-related processes (Lugo et al. 2014).Aberrations in this pathway have been implicated in a range of behavioral and neurochemical abnormalities, including repetitive behaviors, increased anxiety, social interaction difficulties, and disruptions in serotonin transmission (Beaulieu et al. 2009). Moreover, PTEN mutations have been more frequently detected in individuals with global developmental delays and intellectual impairments (Varga et al. 2009). Structural alterations such as hippocampal hypertrophy and increased dendritic complexity have also been observed in PTEN-deficient models (Schumann et al. 2004) which may interfere with the fine-tuning of synaptic networks critical for learning. Given that the hippocampus plays a central role in working memory, information encoding, and retrieval, PTEN-related signaling abnormalities may contribute to the cognitive deficits seen in SLD. Impaired regulation of synaptogenesis and dendritic growth may disrupt neural circuitry involved in language processing, mathematical reasoning, and executive functioning, potentially underlying the academic difficulties characteristic of SLD.

This disruption can lead to the characteristic behavioral and cognitive deficits associated with the disorder. In our previous studies, we have demonstrated the roles of epigenetic regulators in neurodevelopmental processes in individuals with SLD. In our previous study, where the upregulation of MNK1 and SYNGAP1 genes was detected, attention was drawn to the effects of SP and translational control mechanisms on learning processes (Isik et al. 2025). However, the significant increases observed in the expression levels of dyslexia-related genes suggested that epigenetic dysregulation in SLD may affect large-scale genetic networks (Bayyurt et al. 2024). This study support the fact that the significant increase in MeCP2 and decrease in PTEN in our current study are parallel to these previously described epigenetic changes and that a large epigenetic reprogramming may be involved in the molecular basis of SLD. In particular, MeCP2 is a transcriptional regulator that can affect genes directly related to synaptic regulation, such as SYNGAP1, suggesting that it may cause cognitive dysfunction. The findings obtained from regression analysis revealed that PTEN gene expression is affected by some environmental and clinical factors (Table 3). It was observed that drug use during pregnancy significantly decreased PTEN expression, while disease severity increased PTEN levels. This may suggest that pharmacological agents exposed during the prenatal period may suppress the PTEN gene through epigenetic mechanisms. On the other hand, the positive relationship between disease severity and PTEN expression suggests that this gene may play an adaptive or compensatory role in the pathophysiology of ND. The fact that no significant finding was obtained in terms of MeCP2 gene expression may suggest that this gene may be more resistant to environmental factors or may be affected through different interaction networks.

In conclusion, this study shows that MeCP2 gene is significantly upregulated and PTEN gene is significantly downregulated in individuals with SLD, suggesting that learning disabilities may be related to epigenetic-based molecular mechanisms. Considering the regulatory role of MeCP2 on transcriptional control and SP and the effects of PTEN on cellular survival and neuroprotection, the imbalance in the expression of these two genes may be one of the causes of neurobiological disorders in SLD. The findings show that MeCP2 and PTEN do not only play a role in the molecular course of the disease but can also be evaluated as biomarkers for early diagnosis and therapeutic targets for individualized treatment approaches in the future. In this context, our study can be seen as an important starting point for the development of new epigenetic-based approaches in the diagnosis and treatment of SLD.

## Data Availability

No datasets were generated or analysed during the current study.

## References

[CR1] Amador-Arjona A, Cimadamore F, Huang C-T et al (2015) SOX2 primes the epigenetic landscape in neural precursors enabling proper gene activation during hippocampal neurogenesis. Proc Natl Acad Sci U S A 112:E1936-1945. 10.1073/pnas.1421480112 25825708 10.1073/pnas.1421480112 PMC4403144

[CR2] Baohan A, Ikrar T, Tring E et al (2016) Pten and EphB4 regulate the establishment of perisomatic inhibition in mouse visual cortex. Nat Commun 7:12829. 10.1038/ncomms12829 27611660 10.1038/ncomms12829 PMC5023968

[CR3] Bayyurt B, Şahin NÖ, Işık CM (2024) Investigation of association between expression of DYX1C1, KIAA0319, and ROBO1 genes and specific learning disorder in children and adolescents. J Mol Neurosci 74:109. 10.1007/s12031-024-02288-2 39542997 10.1007/s12031-024-02288-2

[CR4] Beaulieu J-M, Gainetdinov RR, Caron MG (2009) Akt/GSK3 signaling in the action of psychotropic drugs. Annu Rev Pharmacol Toxicol 49:327–347. 10.1146/annurev.pharmtox.011008.145634 18928402 10.1146/annurev.pharmtox.011008.145634

[CR5] Benetatos J, Bennett RE, Evans HT et al (2020) PTEN activation contributes to neuronal and synaptic engulfment by microglia in tauopathy. Acta Neuropathol 140:7–24. 10.1007/s00401-020-02151-9 32236736 10.1007/s00401-020-02151-9 PMC7300099

[CR6] Blackman MP, Djukic B, Nelson SB, Turrigiano GG (2012) A critical and cell-autonomous role for MeCP2 in synaptic scaling up. J Neurosci 32:13529–13536. 10.1523/JNEUROSCI.3077-12.2012 23015442 10.1523/JNEUROSCI.3077-12.2012 PMC3483036

[CR7] Busch RM, Srivastava S, Hogue O et al (2019) Neurobehavioral phenotype of autism spectrum disorder associated with germline heterozygous mutations in PTEN. Transl Psychiatry 9:253. 10.1038/s41398-019-0588-1 31594918 10.1038/s41398-019-0588-1 PMC6783427

[CR8] Cheng J, Huang M, Zhu Y et al (2014) SUMOylation of MeCP2 is essential for transcriptional repression and hippocampal synapse development. J Neurochem 128:798–806. 10.1111/jnc.12523 24188180 10.1111/jnc.12523

[CR9] Clipperton-Allen AE, Page DT (2014) Pten haploinsufficient mice show broad brain overgrowth but selective impairments in autism-relevant behavioral tests. Hum Mol Genet 23:3490–3505. 10.1093/hmg/ddu057 24497577 10.1093/hmg/ddu057

[CR10] Fortes IS, Paula CS, Oliveira MC et al (2016) A cross-sectional study to assess the prevalence of DSM-5 specific learning disorders in representative school samples from the second to sixth grade in Brazil. Eur Child Adolesc Psychiatry 25:195–207. 10.1007/s00787-015-0708-2 25925785 10.1007/s00787-015-0708-2

[CR11] Frazier TW, Embacher R, Tilot AK et al (2015) Molecular and phenotypic abnormalities in individuals with germline heterozygous PTEN mutations and autism. Mol Psychiatry 20:1132–1138. 10.1038/mp.2014.125 25288137 10.1038/mp.2014.125 PMC4388743

[CR12] Gallent EA, Steward O (2018) Neuronal PTEN deletion in adult cortical neurons triggers progressive growth of cell bodies, dendrites, and axons. Exp Neurol 303:12–28. 10.1016/j.expneurol.2018.01.005 29337147 10.1016/j.expneurol.2018.01.005 PMC5864555

[CR13] Gambino F, Khelfaoui M, Poulain B et al (2010) Synaptic maturation at cortical projections to the lateral amygdala in a mouse model of Rett syndrome. PLoS ONE 5:e11399. 10.1371/journal.pone.0011399 20625482 10.1371/journal.pone.0011399 PMC2896423

[CR14] Garro-Martínez E, Fullana MN, Florensa-Zanuy E et al (2021) mTOR knockdown in the ınfralimbic cortex evokes a depressive-like state in mouse. Int J Mol Sci 22:8671. 10.3390/ijms22168671 34445375 10.3390/ijms22168671 PMC8395521

[CR15] Grizzle R (2011) Wechsler ıntelligence scale for children, Fourth Edition. In: Goldstein S, Naglieri JA (eds) Encyclopedia of Child Behavior and Development. Springer, US, Boston, MA, pp 1553–1555

[CR16] Isik CM, Bayyurt EBT, Sahin NO (2025) The MNK-SYNGAP1 axis in specific learning disorder: gene expression pattern and new perspectives. Eur J Pediatr 184:260. 10.1007/s00431-025-06089-6 40108041 10.1007/s00431-025-06089-6 PMC11922980

[CR17] Kaner S, Buyukozturk S, Iseri E (2013) Conners parent rating scale-revised short Turkish standardization study Arch. Neuropsychiatr 50:100–109

[CR18] Kath C, Goni-Oliver P, Müller R et al (2018) PTEN suppresses axon outgrowth by down-regulating the level of detyrosinated microtubules. PLoS ONE 13:e0193257. 10.1371/journal.pone.0193257 29617365 10.1371/journal.pone.0193257 PMC5884485

[CR19] Kaufman J, Birmaher B, Brent D et al (1997) Schedule for affective disorders and schizophrenia for school-age children-present and lifetime version (K-SADS-PL): initial reliability and validity data. J Am Acad Child Adolesc Psychiatry 36:980–988. 10.1097/00004583-199707000-00021 9204677 10.1097/00004583-199707000-00021

[CR20] Kramer JM (2013) Epigenetic regulation of memory: implications in human cognitive disorders. Biomol Concepts 4:1–12. 10.1515/bmc-2012-0026 25436561 10.1515/bmc-2012-0026

[CR21] Kwak Y-D, Ma T, Diao S et al (2010) NO signaling and S-nitrosylation regulate PTEN inhibition in neurodegeneration. Mol Neurodegener 5:49. 10.1186/1750-1326-5-49 21067594 10.1186/1750-1326-5-49 PMC2992530

[CR22] Liu X, Ma J, Ding G et al (2021) Microglia polarization from M1 toward M2 phenotype ıs promoted by astragalus polysaccharides mediated through ınhibition of miR-155 in experimental autoimmune encephalomyelitis. Oxid Med Cell Longev 2021:5753452. 10.1155/2021/5753452 34976303 10.1155/2021/5753452 PMC8720009

[CR23] Lugo JN, Smith GD, Arbuckle EP et al (2014) Deletion of PTEN produces autism-like behavioral deficits and alterations in synaptic proteins. Front Mol Neurosci 7:27. 10.3389/fnmol.2014.00027 24795561 10.3389/fnmol.2014.00027 PMC3997048

[CR24] Lyu J-W, Yuan B, Cheng T-L et al (2016) Reciprocal regulation of autism-related genes MeCP2 and PTEN via microRNAs. Sci Rep 6:20392. 10.1038/srep20392 26843422 10.1038/srep20392 PMC4740767

[CR25] Lyu Y, Bai L, Qin C (2019) Long noncoding RNAs in neurodevelopment and Parkinson’s disease. Animal Model Exp Med 2:239–251. 10.1002/ame2.12093 31942556 10.1002/ame2.12093 PMC6930994

[CR26] Maezawa I, Jin L-W (2010) Rett syndrome microglia damage dendrites and synapses by the elevated release of glutamate. J Neurosci 30:5346–5356. 10.1523/JNEUROSCI.5966-09.2010 20392956 10.1523/JNEUROSCI.5966-09.2010 PMC5533099

[CR27] Margari L, Buttiglione M, Craig F et al (2013) Neuropsychopathological comorbidities in learning disorders. BMC Neurol 13:198. 10.1186/1471-2377-13-198 24330722 10.1186/1471-2377-13-198 PMC3878726

[CR28] Mellén M, Ayata P, Dewell S et al (2012) MeCP2 binds to 5hmC enriched within active genes and accessible chromatin in the nervous system. Cell 151:1417–1430. 10.1016/j.cell.2012.11.022 23260135 10.1016/j.cell.2012.11.022 PMC3653293

[CR29] Narayanan RP, Jose LK, Ramanujan JM, Vidyadharan V (2018) Prevalence and correlates of depressive symptoms among children with specific learning disorder attending a tertiary care centre. Kerala Journal of Psychiatry 31:. 10.30834/KJP.31.2.2018.154

[CR30] Nettles SA, Ikeuchi Y, Lefton KB et al (2023) MeCP2 represses the activity of topoisomerase IIβ in long neuronal genes. Cell Rep 42:113538. 10.1016/j.celrep.2023.113538 38096051 10.1016/j.celrep.2023.113538 PMC10844882

[CR31] Nguyen MVC, Du F, Felice CA et al (2012) MeCP2 is critical for maintaining mature neuronal networks and global brain anatomy during late stages of postnatal brain development and in the mature adult brain. J Neurosci 32:10021–10034. 10.1523/JNEUROSCI.1316-12.2012 22815516 10.1523/JNEUROSCI.1316-12.2012 PMC3461266

[CR32] Nolan SO, Jefferson TS, Reynolds CD et al (2019) Neuronal deletion of phosphatase and tensin homolog results in cerebellar motor learning dysfunction and alterations in intracellular signaling. NeuroReport 30:556–561. 10.1097/WNR.0000000000001241 30920436 10.1097/WNR.0000000000001241 PMC6506221

[CR33] Opazo P, Watabe AM, Grant SGN, O’Dell TJ (2003) Phosphatidylinositol 3-kinase regulates the induction of long-term potentiation through extracellular signal-related kinase-independent mechanisms. J Neurosci 23:3679–3688. 10.1523/JNEUROSCI.23-09-03679.2003 12736339 10.1523/JNEUROSCI.23-09-03679.2003 PMC6742185

[CR34] Petretto DR, Masala C (2017) Dyslexia and specific learning disorders: new ınternational diagnostic criteria. J Child Dev Disord 03: 10.4172/2472-1786.100057

[CR35] Sahoo MK, Biswas H, Padhy SK (2015) Psychological co-morbidity in children with specific learning disorders. J Family Med Prim Care 4:21–25. 10.4103/2249-4863.152243 25810984 10.4103/2249-4863.152243 PMC4367000

[CR36] Sanfilippo J, Ness M, Petscher Y et al (2020) Reintroducing dyslexia: early ıdentification and ımplications for pediatric practice. Pediatrics 146:e20193046. 10.1542/peds.2019-3046 32576595 10.1542/peds.2019-3046 PMC7329249

[CR37] Schumann CM, Hamstra J, Goodlin-Jones BL et al (2004) The amygdala is enlarged in children but not adolescents with autism; the hippocampus is enlarged at all ages. J Neurosci 24:6392–6401. 10.1523/JNEUROSCI.1297-04.2004 15254095 10.1523/JNEUROSCI.1297-04.2004 PMC6729537

[CR38] Spinelli L, Black FM, Berg JN et al (2015) Functionally distinct groups of inherited PTEN mutations in autism and tumour syndromes. J Med Genet 52:128–134. 10.1136/jmedgenet-2014-102803 25527629 10.1136/jmedgenet-2014-102803 PMC4316932

[CR39] Ünal F, Öktem F, Çetin Çuhadaroğlu F et al (2019) Reliability and validity of the schedule for affective disorders and schizophrenia for school-age children-present and lifetime version, DSM-5 November 2016-Turkish Adaptation (K-SADS-PL-DSM-5-T). Turk Psikiyatri Derg 30:42–50 31170306

[CR40] Varga EA, Pastore M, Prior T et al (2009) The prevalence of PTEN mutations in a clinical pediatric cohort with autism spectrum disorders, developmental delay, and macrocephaly. Genet Med 11:111–117. 10.1097/GIM.0b013e31818fd762 19265751 10.1097/GIM.0b013e31818fd762

[CR41] Vogt D, Cho KKA, Lee AT et al (2015) The parvalbumin/somatostatin ratio is increased in Pten mutant mice and by human PTEN ASD alleles. Cell Rep 11:944–956. 10.1016/j.celrep.2015.04.019 25937288 10.1016/j.celrep.2015.04.019 PMC4431948

[CR42] Weiss E, Akimjaková B, Paľa G, Biryukova YN (2023) Methods of re-education of specific learning disorders. Journal of Education Culture and Society 14:185–197

[CR43] Wu D, Li R (2022) Genetic analysis of neurodevelopmental disorders in children. Front Child Adolesc Psychiatry 1:987339. 10.3389/frcha.2022.987339 39817275 10.3389/frcha.2022.987339 PMC11731956

[CR44] Xu K, Yang Y, Ding J et al (2024) Spatially precise genetic engineering at the electrode-tissue ınterface. Adv Mater 36:e2401327. 10.1002/adma.202401327 38692704 10.1002/adma.202401327

[CR45] Zhao Y-T, Goffin D, Johnson BS, Zhou Z (2013) Loss of MeCP2 function is associated with distinct gene expression changes in the striatum. Neurobiol Dis 59:257–266. 10.1016/j.nbd.2013.08.001 23948639 10.1016/j.nbd.2013.08.001 PMC3790640

[CR46] Zhong X, Li H, Chang Q (2012) MeCP2 phosphorylation is required for modulating synaptic scaling through mGluR5. J Neurosci 32:12841–12847. 10.1523/JNEUROSCI.2784-12.2012 22973007 10.1523/JNEUROSCI.2784-12.2012 PMC3474205

